# A novel BMSN (biologically synthesized mesoporous silica nanoparticles) material: synthesis using a bacteria-mediated biosurfactant and characterization

**DOI:** 10.1039/d1ra05852e

**Published:** 2021-10-06

**Authors:** Raju Kumar Sharma, Shau-Chun Wang, Jyoti Prakash Maity, Pritam Banerjee, Gobinda Dey, Yi-Hsun Huang, Jochen Bundschuh, Ping-Gune Hsiao, Tsung-Hsien Chen, Chien-Yen Chen

**Affiliations:** Department of Chemistry and Biochemistry, National Chung Cheng University 168 University Road, Min-Hsiung Chiayi County 62102 Taiwan; Department of Earth and Environmental Sciences, National Chung Cheng University 168 University Road, Min-Hsiung Chiayi County 62102 Taiwan chien-yen.chen@oriel.oxon.org; Department of Chemistry, School of Applied Sciences, KIIT Deemed to be University Bhubaneswar Odisha 751024 India jyotiprakash.maityfch@kiit.ac.in jyoti_maity@yahoo.com; Department of Biomedical Science, Graduate Institute of Molecular Biology, National Chung Cheng University 168 University Road, Min-Hsiung Chiayi County 62102 Taiwan; UNESCO Chair on Groundwater Arsenic within the 2030 Agenda for Sustainable Development, University of Southern Queensland (USQ) West Street Toowoomba QLD 4350 Australia; Department of Internal Medicine, Ditmanson Medical Foundation Chia-Yi Christian Hospital Chiayi City Taiwan

## Abstract

Mesoporous materials (MMs) have recently been applied as advanced nanomaterials in different fields (separation, catalysis, adsorption *etc.*). Synthesis of MMs by chemical surfactants is not ecofriendly. This study focused on the biological synthesis of a MM by sol–gel method, using a *Bacillus subtilis* BBK006-mediated surfactant (template) and a precursor (TEOS). The biologically synthesized mesoporous silica nanoparticles (BMSN) were formed at calcination temperatures of 450–600 °C. The BMSN comprise Si and O elements with specific weights of 56.09% and 42.13% respectively, where the atomic% was detected to be 41.79% and 55.10%, respectively. The phase identity of the synthesized particles (61–300 nm uniform spherical shape; surface area: 8.2616 m^2^ g^−1^; pore diameter at 550 °C: 14.8516 nm) was confirmed with wide-angle XRD (10°–81°). A typical type IV isotherm was exhibited (BET curves) following IUPAC nomenclature and confirmed the mesoporous nature. The green-synthesized biosurfactant-mediated BMSN is an environmentally promising material to apply in biomedical science (*e.g.*, antimicrobial activity, drug delivery, CMC, anticancer activity) and oil spill management.

## Abstract 2

Synthesis of a novel BMSN material using bacteria-mediated biosurfactant and their characterization.

## Graphical abstract

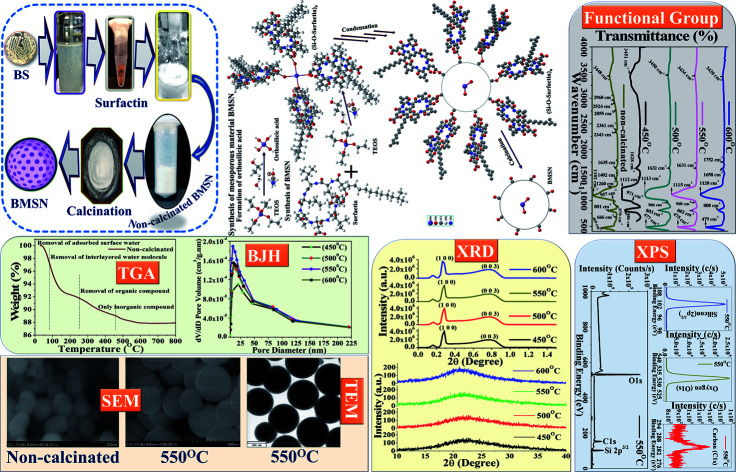

## 1. Introduction

MMs have been receiving wide interest in the field of material science owing to their potential utilization as adsorbents, controlled drug delivery systems, sensors, catalysts in chemical reactions, cosmetics *etc.*^1–3^ Primarily, the long-chain carbon surfactants and templating agents are responsible for the pore size variation of MMs.^4^ Chemically and morphologically, the MMs have revealed a potential particle with highly coordinated spherical shape (2–50 nm pore diameter).^5^ Technologically, MMs are synthesized through numerous methods (template-assisted, sol–gel, microwave-assisted, and chemical etching),^6^ which are generally time-consuming and require high temperature and pressure.^7^

In particular, the sol–gel method is popular as it is easy, less time-consuming (compared to other methods), has a low operational temperature, and is a more expedient process for synthesizing MMs.^8^ Numerous chemical surfactants (*e.g.*, CTAB, P123, F127, CTAC) play an important role in the synthesis of MMs with high specific surface area and desired shape comprising well defined structural morphology.^7,9,10^ C_19_H_42_N^+^OH^−^/Cl^−^ (a chemical surfactant) was used for the first time to synthesize the mesoporous spherical material MCM-41.^11^ With the progress of science and technology, cetyltrimethylammonium bromide (CTAB) surfactant has been applied to synthesize the mesoporous material MCM-41 due to its very low/no toxicity^12^ and efficiency at very low concentrations (≤1 μM);^12^ however, chemical surfactants (*e.g.* CTAB) still exhibit toxicity to living organisms at higher concentrations (>1 μM), such as cytotoxicity (human epidermal keratinocytes in HaCaT cells, lung fibroblasts in CRL-1490 cells) and chronic and subacute toxicity (male/female rats).^12–14^

In recent years, biosurfactants have received increasing interest in different research arenas (heavy metals removal, pharmaceuticals, food, detergents, petroleum, cosmetics, agriculture, *etc.*) due to their low toxicity,^15–18^ ecofriendly nature, low cost and easy synthesis. Microbiologically, biosurfactants are produced from microorganisms with different principal carbon sources (carbohydrates, industrial/domestic wastewater, starch-containing substrates, vegetable oils, renewable resources, *etc.*).^19,20^ Currently, a significant percentage of biosurfactant production takes place in Europe [Germany (Biotensidon, Evonik), France, Italy, Belgium (Ecover), UK, *etc.*], as well as India, China, Japan, Thailand, Brazil, and the USA (Jeneil Biotech) in the global market,^21^ where biosurfactants are mainly utilized in personal care, agriculture, the food industry, detergents, *etc.* The production of biosurfactants is now easy and effective due to advances with analytical instruments in industry that allow biosurfactants to be conveniently extracted from various microbes.^15,16,22,23^ Apart from these advantages, biosurfactants are biodegradable, environmentally friendly, economical, and renewable substrates.^24^ Moreover, the physical characteristics of biosurfactants are mainly dependent on the temperature and pH.^24^ Thus, in nanobiotechnology, natural substrates are used as precursors for synthesizing microbial-based nanoparticles to minimize the hindrance (*e.g.* reduce the toxicity and increase the ecofriendliness).

In the present study, a *Bacillus subtilis* BBK006-derived biosurfactant was used as the biological template to synthesize a novel material (BMSN), which is a key objective for improving the economic and environmental aspects of nanobiotechnology. A sol–gel process (bottom-up approach) with different calcining temperatures was used to synthesize the BMSN. The physicochemical characteristics of the BMSN were investigated in terms of crystal structure, chemical bonding, surface adsorption, absorptivity, morphology, pore shape and size, *etc.*

## 2. Materials and methods

### 2.1 Chemicals and reagents

The biosurfactant was extracted from *Bacillus subtilis* BBK006 (a non-pathogenic bacterium, collected from a fuel-contaminated site).^15,16,22,25,26^ The chemical reagents Na_2_HPO_4_ (98% purity), KH_2_PO_4_ (99.5% assay), NaCl (99.9% assay), NH_4_Cl (99.5% purity), MgSO_4_·7H_2_O (99.8% assay), CaCl_2_ (76.07% assay) and glucose (99.9% purity) were used to prepare the M9 medium for bacterial growth.^15,16,22,25,26^ The material was synthesized using 25 wt% of aqueous ammonia, ethyl alcohol (99.5% assay), tetraethyl orthosilicate (TEOS) (99.0% purity; Fluka) and methanol. All chemical reagents were used without further purification.

### 2.2 Preparation of media and bacterial culture

The *Bacillus subtilis* BBK006 was grown in M9 broth media with the following composition: 6.78 g L^−1^ of Na_2_HPO_4_, 3.0 g L^−1^ of KH_2_PO_4_, 0.5 g L^−1^ of NaCl, 1.0 g L^−1^ of NH_4_Cl, 0.493 g L^−1^ of MgSO_4_·7H_2_O, and 0.011 g L^−1^ of CaCl_2_.^15,16,22,25,26^ All of these chemicals were entirely dissolved in the double deionized water separately to avoid the formation of precipitation on the surface of the transparent glass reagent bottle during the sterilization process. The dissolved components were transferred to a 1 L transparent glass reagent bottle and made up to the mark to maintain the balanced concentration. The prepared broth media was wrapped with aluminium foil for sterilization (autoclave at 121 °C for 20 min at 15 atm). The sterilized media was cooled down and placed in laminar flow hood for inoculation with *Bacillus subtilis* BBK006. 0.4% glucose was added to the sterilized broth media in laminar flow hood (to avoid external bacterial contamination). A small amount (2 μL at ∼1.2 OD) of bacteria was inoculated for growth.

The flask containing the inoculated bacterial media was placed in an orbital shaking incubator (JSL-530) at 37 °C (200 rpm) and incubated for 24 h to increase the bacterial count per volume. The highest growth of bacteria was estimated as ∼1.2 OD (optical density in CFU mL^−1^ at 600 nm wavelength) by UV-vis-spectrophotometer (UV-vis, PRO-739, Prema), and was used for biosurfactant production. The scan rate of the ultraviolet-visible spectrophotometer is 2500 nm min^−1^ with accuracy of ±1.2 nm over the wavelength range of 320–1100 nm using a halogen bulb as the light source.

### 2.3 Production of biosurfactants

Bacterial population growth plays an important role in producing biosurfactants since the production rate is proportional to the amount/number of bacteria. The biosurfactant was produced following the procedure of Maity *et al.*^16^ Highly populated bacterial media (∼1.2 OD_600_) was centrifuged at 10 000 rpm (12 000 × *g*) for 15 min, at 25 °C (Himac CR-22G, R20A2 Rotor, Hitachi, Japan) to collect the supernatant (without bacterial cells). The collected supernatant was adjusted to pH < 2 using 5 M hydrochloric acid (HCl) to precipitate the biosurfactant properly by incubating overnight (12 h). After precipitation, the biosurfactant was collected by centrifugation at 5000 rpm for 12 min at room temperature and the precipitated biosurfactant was then dried to a powder using a precision oven (JA-72) set to 65 °C for 24 h. Finally, the biosurfactant was obtained as a solid light brown powder, which was used to synthesize the MM.

### 2.4 Synthesis of mesoporous material

The MM was synthesized following a novel procedure. Initially, 0.57 g of biosurfactant was dissolved in 50 mL of double deionized water, and stirred continuously until a clear solution was obtained. 11 mL of 25 wt% ammonia and 76 mL of ethanol were added to the clear solution simultaneously. At the same time, 10 mL of TEOS was also added dropwise to the mixture whilst stirring the solution. The mixed solution turned into a milky gel form due to the hydrolysis of TEOS. Gradually, the mixed solution was uninterruptedly stirred for ∼1 h 45 min to complete the hydrolysis of TEOS. A white precipitate was observed in the mixture and was separated by centrifugation at 5000 rpm for 10 min, (room temperature, 25 °C). The precipitate was washed with double deionized water and methanol thrice, and then dried at 70 °C for 24 h. The dried material was finally calcined at different temperatures (450 °C, 500 °C, 550 °C, and 600 °C) in a muffle furnace to produce the MMs.

### 2.5 Characterizations of synthesized particles

#### 2.5.1 X-ray diffraction (XRD) of the synthesized material

The crystallographic structure, phase composition, and physical properties of the materials were determined by XRD following the procedure of Maity *et al.*^27^ The small-angle powder X-ray diffraction pattern was recorded using a Vantec-2000 detector with an exposure time of 3600 s per frame, with Ni-filtered KFL Cu Kα radiation (Bruker AXS Gmbh instrument, Nanostar U System, Karlsruhe, Germany), an operating current of 0.65 mA and a voltage of 45 kV with a 2*θ* range of 0.1 to 5°. The wide-angle X-ray diffraction patterns were recorded using Ni-filtered Cu Kα radiation with an operating current of 10 mA, voltage of 30 kV and a scintillation counter detector (Bruker MeaSrv-D2-205680 Phaser instrument, *λ* = 1.540600 nm). The data were collected in 2*θ* angle with the range of 10–81° at elevated temperature (3 °C per minute) and a step size of 0.020135°.

#### 2.5.2 Identification of functional groups in the synthesized materials by FTIR

The functional groups of the biosurfactant-mediated raw material and the synthesized/calcinated MMs were determined using Fourier transform infrared spectroscopy (FTIR, Bruker optics-2141 Vertex-70V in an RT-DLaTGS sample compartment) following the procedure of Maity *et al.*^15^ The functional groups were determined with KBr powder (KBr/sample weight ratio = 20 : 1) in the range of 4000–400 cm^−1^.

#### 2.5.3 Elemental quantification of synthesized materials by XPS

High-resolution X-ray photoelectron spectroscopy (XPS, PHI Hybrid Quantera, USA) was used for the elemental quantification of the synthesized materials. Al Kα radiation was used in the XPS as a monochromatic X-ray beam of light with 49.3 W, 45.0°, and 280.00 eV.

#### 2.5.4 Determination of absorption of synthesized materials by UV-vis-NIR

The absorption of the synthesized materials was determined by ultraviolet-visible/near-infrared spectrometry (UV-vis-NIR; HITACHI U4100, Japan). The UV-vis-NIR spectrometer recorded (*λ* range = 200–2600 nm) the reflectance spectra of the samples with BaSO_4_ as a reference sample and constant temperature using cooling Pbs Gain detector.

#### 2.5.5 Assessment of thermal stability of synthesized materials by TG-DTA

The degradation process and thermal stability of the synthesized materials were investigated using a TG-DTA thermogravimetric analyzer (PerkinElmer STA 6300 instrument, USA).^27^ The analysis was carried out in the range of 30–800 °C with a heating rate of 10 °C min^−1^ under a nitrogen atmosphere.

#### 2.5.6 Assessment of morphology of synthesized materials by FE-SEM and HR-TEM-EDX

The surface morphology of the materials was determined following the procedure of Maity *et al.* using a field emission-scanning electron microscope (FE-SEM) (Hitachi S4800-I instrument, Japan) with a coating of Pt in the operating range of 0.1–30 kV.^16^ The particle size and morphology of the synthesized materials were determined using a high-resolution transmission electron microscope (HR-TEM) (JEOL JEM-2010, Taiwan) with an acceleration voltage of 200 kV. HR-TEM is carried the electron gun filament LaB_6_. The elemental analysis was also carried out by HR-TEM/EDX with the same analytical instrument.

#### 2.5.7 Assessment of surface properties (pore volume, pore diameter and specific surface area) of the synthesized materials by BET

The pore volume, pore diameter and specific surface area were studied using the Brunauer, Emmett, and Teller method with a surface area analyzer instrument (BET, Micromeritics, ASAP 2020 PLUS, USA). The surface analysis was performed in the presence of liquid nitrogen at 77 K.^16^

## 3. Results and discussion

### 3.1 Properties of synthesized materials

#### 3.1.1 Crystallographic properties

**Fig. 1:**
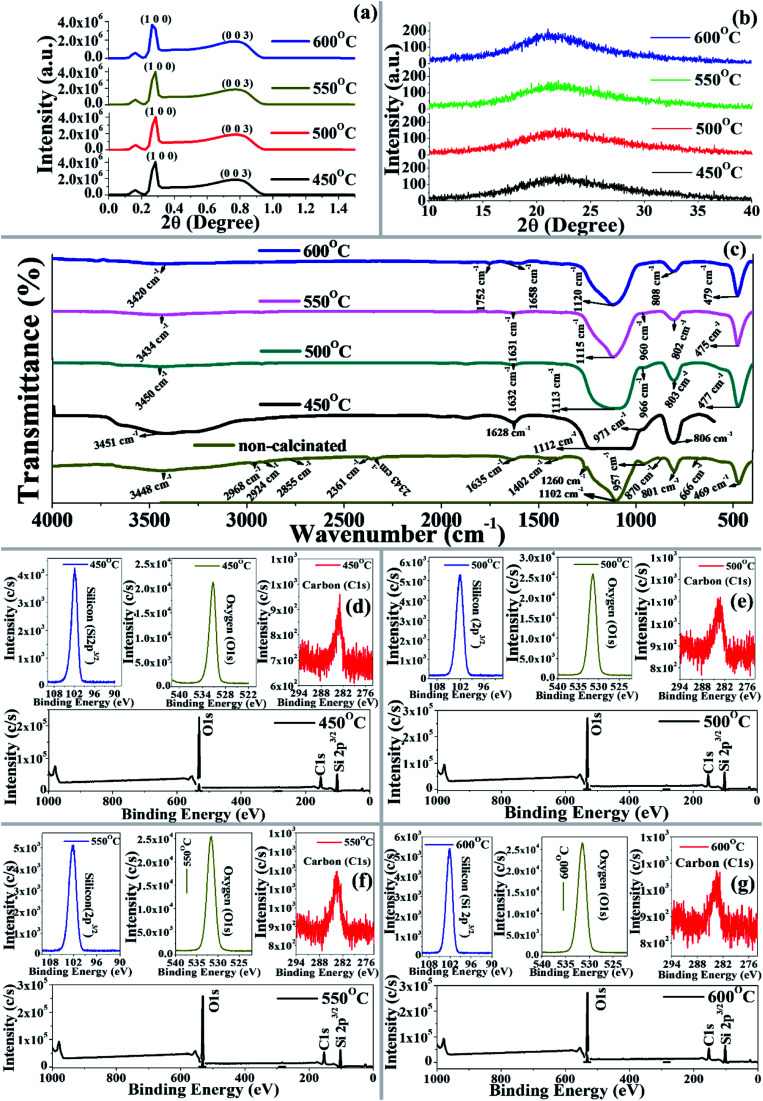
(a) Low-angle and (b) wide-angle X-ray diffraction patterns of BMSN prepared at different calcination temperatures (450 °C, 500 °C, 550 °C, and 600 °C). (c) Fourier transform infrared spectra of synthesized BMSN material: non-calcinated and calcinated (450 °C, 500 °C, 550 °C, and 600 °C). X-ray photoelectron spectra of BMSN synthesized at different calcination temperatures: 450 °C (d), 500 °C (e), 550 °C (f), and 600 °C (g).

The phase composition and crystallinity of the synthesized materials (BMSN) were determined using both small-angle and wide-angle XRD (Fig. 1a and b). The two characteristic peaks assigned as the *d*_100_ and *d*_003_ planes in the low-angle XRD spectrum (Fig. 1a) are ascribed to the two-dimensional hexagonal mesoporous character, where diffraction angles and intensities at each calcinated temperature (*i.e.* 450 °C, 500 °C, 550 °C, and 600 °C) were found to be almost the same, which is similar to the findings of Wu *et al.*^28^ The unit cell diameter of BMSN was observed to be higher (unit cell diameter 62.18 Å) than the chemical surfactant-based synthesized material (unit cell diameter < 50 Å) owing to efficient collaboration between the precursor and the biosurfactants.^5,29^ The phase composition and crystallinity of the synthesized materials (BMSN) were determined using wide-angle XRD (Fig. 1b). In the present study, broad diffraction peaks were observed at 2*θ* of 21.72 (*d*_100_), 21.95 (*d*_100_), 22.59 (*d*_100_), and 21.03 (*d*_100_) for the calcination temperatures of 450 °C, 500 °C, 550 °C, and 600 °C, respectively (Fig. 1b), which indicates that the obtained particles are an amorphous mesoporous silica material, and these results are comparable with the finding of Sarawade *et al.*^30^ Therefore, the 2*θ* of the BMSN is in the range of 20–25°, which is characteristic of amorphous mesoporous silica^31^ and the calcination temperature does not have a significant effect on the phase composition. Furthermore, the wide-angle XRD-diffraction peaks of synthesized material were found to be comparable to those of chemical surfactant-based MMs.^29^ However, the low-angle XRD showed diffraction peaks with very low diffraction angles (Fig. 1a–d) as compared to the organic surfactant-based materials, where the decrease of diffraction angle could be dependent upon the amount of silica in the materials.^32^

#### 3.1.2 Functional group elucidation

The FTIR spectral bands of the functional groups were revealed within the range of 4000–400 cm^−1^ (Fig. 1c). In the present study, a broad-band peak was observed at around 3445 cm^−1^ for both the non-calcinated and calcinated materials (450–600 °C), which represents the stretching vibration of a hydroxyl group (2OH).^33^

In the non-calcinated synthesized material, the spectral bands at 2968, 2924 and 2855 cm^−1^ were assigned as –CH_2_ group stretching vibrations, which are comparable with the findings of Huo *et al.*^33^ The multiple bands at 2361 and 2343 cm^−1^ appeared due to N–H group stretching vibration. Strong, medium and numerous very weak intensity bands in the range of 1635–1558 cm^−1^ were assigned to N–H, C=O, NO_2_ and O–H groups, which indicates the appearance of the biosurfactant in the synthesized material. Weak and medium intense peaks were also observed in the BMSN at 1402 and 1456 cm^−1^ for O–H and ring groups. A less intense band at 870 cm^−1^ represents the C–H and C–O–C functional groups with different modes of vibration (Table 1).

**Table 1:** The functional groups of biologically synthesized mesoporous silica nanoparticles (BMSN)

Wavenumber (cm^−1^) before calcination	Wavenumber (cm^−1^) after calcination	Bond	Intensity	Mode	Notes or comments
3700–3200		O–H	M	STR	Broad peak
3450–3442		O–H	S	STR	—
3500–3300		N–H	W	STR	Secondary amine
3500–3422		N–H	M	ASY_STR	—
3500–3300		N–H	M	STR	—
2800–2000		N–H	M	STR	Often multiple bands
1680–1630		C=O	S	STR	Amide I
1650–1550		N–H	VW	DEF	—
1650–1590		H–N–H	MS	DEF	—
1660–1635		C=O	VS	STR	—
1650–1600		NO_2_	S	ASY_STR	—
1640–1580		NH_3_	M	ASY_DEF	—
1640–1632		OH	M	DEF	—
1545–1395		OH	MS	DEF	Series of sharp peaks, R branch
1465–1430		Ring	M	STR	—
1440–1395		O–H	W	DEF	—
1435–1425		Si–Ph	M	STR	—
1410–1310		OH	S	DEF	—
1340–1250		NH_3_	M	SYM_DEF	—
1320–1211		C–O (C–C–COOH)	S	STR	—
1315–1250		C–N	MS	STR	—
1310–1250		C–O	S	STR	—
1310–1210		C–O–C	VS	ASY_STR	—
1305–1200		CNH (–CO–NH–C)	MW	Combination of C–N STR and N–H BEND	Combination amide III
1300–1250		NO_2_	S	SYM_STR	—
1290–1250		C–H	W	BEND	In-plane H bend
1285–1245		CN	M	STR	—
1280–1240		O–C–O	M	STR	—
1280–1250		CH_3_ {Si–CH_3_, Si–(CH_3_)_2_, Si–(CH_3_)_3_}	S	DEF	Sharp peak
1280–1230		C–O–C	M	SYM_STR	Mono-, di-, tri-substituted epoxy ring
1275–1250		C–N (NH–CO–NH)	M	STR	—
1265–1200		C–N & C–O (R–NHCOO–R)	M	STR	Amide IV
1240–1170		C–N	WM	STR	—
1210–1100		C–O	S	STR	Tertiary alcohol
1175–1038		C–O–C	S	STR	Absence of strong band is good
1150–1060		C–O–C	VS	ASY_STR	—
1140–1080		C–N	WM	STR	—
	1130–1090	Si–Ph	S	STR	—
1125–1090		C–O	S	STR	Secondary aliphatic alcohol
	1100–1000	Si–O–Si	VS	STR	Open chain
1110–1090		C–O–C	S	ASY_STR	6 ring ether
1105–1085		C–H	W	BEND	In-plane H bend
	1100–1000	Si–O–C	S	STR	Open chain
	990–945	Si–O–C	S	STR	—
970–920		Si–O	S	STR	—
965–950		Ring	W	DEF	Out-of-plane ring bend
960–900		C–H	M	DEF	1 adjacent H out-of-plane DEF
		C–H	W	DEF	4 adjacent H out-of-plane DEF
950–815		C–O–C	S	DEF	—
		C–O–C	S	ASY_STR	—
880–830		C–H	M	DEF	3 adjacent H out-of-plane DEF
880–805		C–O–C	S	DEF	—
870–860		C–H	S	DEF	1 adjacent H out-of-plane DEF
860–780		C–H	S	DEF	2 adjacent H out-of-plane DEF
850–775		C–O–C	S	DEF	—
820–765		C–H	M	DEF	3 adjacent H out-of-plane DEF
840–790		C–H (CR′R′′=CHR)	S	DEF	CH out-of-plane DEF
	845–800	Si–H	S	WAG	—
	814–800	Si–C	V	STR	—
814–751		N–O (R–O–N=O)	S	STR	—
	565–465	O–H	W	DEF	Series of sharp peaks

A spectral band at ∼1635 cm^−1^ was observed in the non-calcinated and calcinated materials due to bending vibrations of the deformational hydroxyl group (–OH), which is comparable with the findings of Salam *et al.*^34^ Spectral bands at 1102, 1105, 1113, 1115, and 1120 cm^−1^ were observed, representing the existence of asymmetrical vibration of a siloxane group (Si–O–Si) in the materials (non-calcinated and calcinated), which is comparable with previous literature.^35^ Moreover, characteristic peaks at 957, 968, 966, 960 cm^−1^ representing the bending vibrations of a –Si–OH functional group were observed for both the non-calcinated and calcinated materials (except 600 °C), which is similar to previous results.^36^ Interestingly, the spectral band of the Si–OH functional group disappeared at 600 °C calcination temperature due to the loss of hydrogen. A peak at 808 cm^−1^ was observed for the symmetrical stretching vibration of Si–O–Si in the non-calcinated material, whereas the peak shifted to 801, 808, 803, 802, and 808 cm^−1^ for the materials calcinated at 450 °C, 500 °C, 550 °C, and 600 °C, respectively. Furthermore, in the present study, the peaks at 450 cm^−1^ to 810 cm^−1^ were found to be free silica as the Si–O–Si group of synthesized particles due to bending vibrations, which is comparable with the previous results of Salam *et al.*^34^ Thus, in the calcinated synthesized material (450–600 °C), it was clearly indicated that the functional groups –H, C=O, NO_2_ and ring had vanished due to the eradication of the biosurfactants from synthesized material, nevertheless, Si–OH still remained and the synthesized material was designated as BMSN (biologically synthesized mesoporous silica nanoparticles).

#### 3.1.3 Surface composition and electronic interactions

X-ray photoelectron spectroscopy (XPS) provides information about the surface composition, electronic interactions and bonding between two elements. In the BMSN, the detected XPS spectra (Fig. 1d–g) clearly show the specific binding energies (B.E.) of the atomic orbitals (Table 2). The major peaks of the BMSN particles were observed as Si 2p^3/2^, C 1s and O 1s atoms (Fig. 1d–g). As per the XPS spectra, the B.E. of the atomic orbitals mainly depends on the calcination temperature. At 450 °C, the B.E. of the Si 2p^3/2^ orbital was observed as a single peak centered at 101.88 eV (27 atomic%). The B.E. of the Si 2p^3/2^ orbital increased with increasing calcination temperature, with 102.05 and 102.11 eV at 500 °C and 550 °C, respectively. However, the B.E. of the Si 2p^3/2^ orbital at 600 °C (102.03 eV) was smaller than that at 550 °C due to less interaction of Si 2p^3/2^ with oxygen, where the B.E. of the O 1s orbital was detected 531.26, 531.42, 531.57, and 531.47 eV at calcination temperatures of 450 °C, 500 °C, 550 °C, and 600 °C, respectively, which indicated the coupling of silicon with oxygen to generate SiO_4_^4−^ by Si–O linkage, and this result is comparable with the findings of Sterczyńska *et al.*^37^ Likewise, the B.E. of oxygen (O 1s) are intense peaks (Fig. 1d–g) in BMSN, and the oxygen group is further coupled with silicon to form Si–O–Si linkages.^38^ A single highly intense peak of silicon and oxygen was identified at 550 °C with the maximum B.E. and atomic percentage (%), where the highest peak intensity designates strong bonding between silicon and oxygen atoms (Fig. 1f and Table 2). The binding energy of BMSN was comparable with other types of surfactants-based different MMs (*e.g.* MCM-41, MCM-48, and SBA-15).^28,39,40^

**Table 2:** Quantitative analysis of biologically synthesized mesoporous silica nanoparticles (BMSN) calcinated at different temperatures^a^

Sample	Atomic orbital	Binding energy (eV)	Atomic (%)
MCM-41 (450 °C)	Si 2p (2p^3/2^)	101.88 (SiC)	27.0
	O 1s	531.26 (PbS–air 3′-)	71.1
	C 1s	282.99 {C in WC (tungsten carbide)}	1.9
MCM-41 (500 °C)	Si 2p (2p^3/2^)	102.05 {natrolite (Na_2_Al_2_Si_3_O_10_)}	27.7
	O 1s	531.42 (O ds OH^−^)	70.5
	C 1s	283.22 (peak attributed to carbide)	1.8
MCM-41 (550 °C)	Si 2p (2p^3/2^)	102.11 (C–O–Si)	28.0
	O 1s	531.57 (zeolite NaY, NaY)	69.8
	C 1s	283.76 (C_6_H_5_X with X = OCH_3_)	2.2
MCM-41 (600 °C)	Si 2p (2p^3/2^)	102.03 {natrolite (Na_2_Al_2_Si_3_O_10_)}	27.8
	O 1s	531.47 (O in Al metal)	71.5
	C 1s	283.56 (–(CH_2_–CH_2_)_*n*_)	0.7

a Reference for database: http://www.lasurface.com/database/liaisonxps.php.

#### 3.1.4 Energy charge transfer and optical properties

**Fig. 2:**
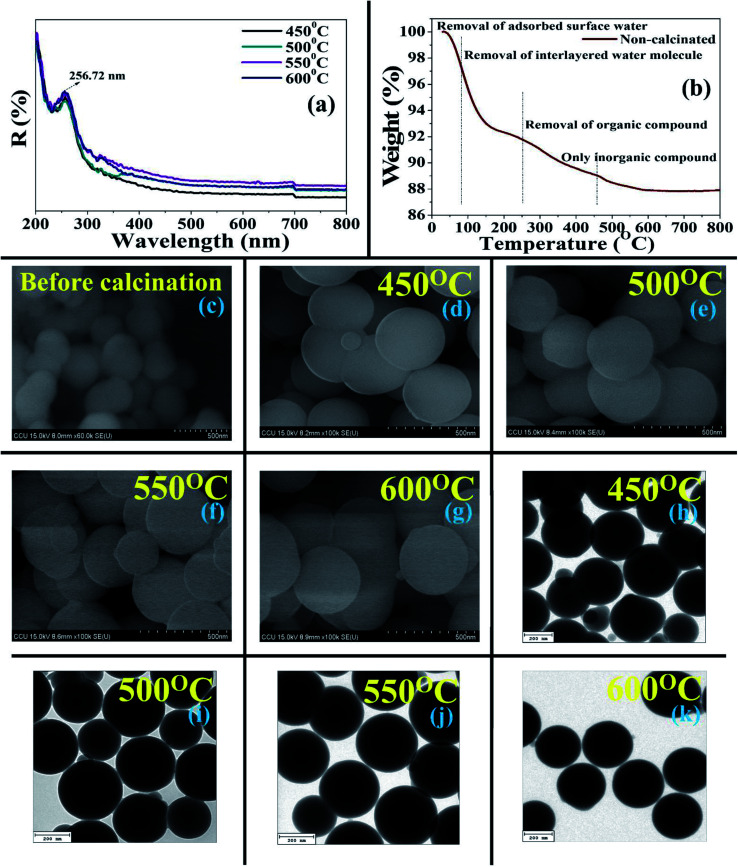
(a) Ultraviolet-visible-near-infrared spectra of synthesized BMSN molecular sieves at different calcination temperatures (450 °C, 500 °C, 550 °C, and 600 °C). (b) Thermogravimetric/differential thermal analysis of synthesized BMSN molecular sieves at different calcination temperatures (450 °C, 500 °C, 550 °C, and 600 °C). Field emission-scanning electron micrographs of BMSN: (c) non-calcinated raw materials, and calcinated at 450 °C (d), 500 °C (e), 550 °C (f), and 600 °C (g). High-resolution transmission electron micrographs of BMSN calcinated at 450 °C (h), 500 °C (i), 550 °C (j), and 600 °C (k).

The spectral properties of a material are a fundamental key to distinguish the coordination environment of the molecules by interpreting the absorption of ultraviolet radiation, where the radiation propagates to transitions flanked by their electronic energy levels. The variation in the absorption spectral band (by UV-vis-NIR) of the material at the various calcination temperatures is shown in Fig. 2a. All of the calcinated materials show an absorption band at 202.62 nm, which indicates the incorporation of molecular sieves with low energy charge transfer. In the present results, the most intense absorption band of each synthesized material was found at 256.72 nm in the ultraviolet region. Furthermore, the spectral intensity of particles amplified from the lower to higher calcinated temperature incessantly, where the intensity was exhibited higher at 550 °C temperature in the ultraviolet region than 600 °C, due to the highest electronic transition between their energy levels. Thus, the absorption was higher in the material synthesized at 550 °C. The detected wavelength of this BMSN was approximately comparable to other kinds of MM (*e.g.* MCM-41, SBA-15, KIT-6 *etc.*).^41,42^

#### 3.1.5 Thermogravimetric analysis

The weight loss (%) by exclusion (organic and inorganic constituents) of surface water, interlayered water molecules, bio-templates, and the establishment of a novel BMSN material at the optimum temperature from the synthesized material are shown in Fig. 2b. The percentage (%) of weight in the synthesized material was already mentioned from 30 to 210 °C due to the eradication of absorbed surface water. Gradually, ∼3% weight loss from the synthesized material was observed at 210–307 °C due to the loss of interlayered water molecules. From 307–468 °C, a weight loss of ∼2% was detected due to the decomposition of a biological compound (the biosurfactant) from the synthesized material. Next, weight loss of ∼1% was observed at 468–600 °C due to the loss of organic compounds from the material as well as the formation of mesoporous silica nanoparticles, whereas no weight loss was observed when the temperature was further increased (>600 °C). Thus, the novel BMSN material was formed at the calcination temperature of 550–600 °C. The total production of BMSN was observed 10.174 mg out of 11.950 mg after the calcination process, where 1.776 mg was removed from the synthesized raw sample. Therefore, the total percentage (%) weight loss and gain was observed to be 12.13 and 87.87% after calcination, respectively. The present finding is comparable with previous research,^43^ which reported a total weight loss of 14.5–16.5% after the formation of a material calcinated at 600–700 °C. Thus, in the present study, a higher production rate of biologically synthesized mesoporous silica nanoparticles (BMSN) was observed.

#### 3.1.6 Morphological and elemental signature

More agglomeration of particles was observed higher in the non-calcinated particles (Fig. 2c), whereas the shape of the calcinated particles (450–600 °C) manifested well-defined morphology with less agglomeration. BMSN are perfectly spherical shaped particles with a more regular arrangement than the non-calcinated materials. The average size distribution was detected within the range of 250–300 nm in diameter (450–600 °C) (Fig. 2d–g). Spherical shaped particles of different types of MMs were reported with well-organized shapes for diverse types of chemical template-based surfactants with average particle diameter in the range of 200–1000 nm,^44^ which indicates that the BMSN particle sizes are smaller/analogous (61–300 nm). Interestingly, the diameter of the BMSN particles was observed to be slightly increased (average particle diameter 264 nm) at 600 °C temperature (Fig. 2g). Overall, the particle size was instituted at an average diameter of 280 nm with a range of 61–300 nm. Thus, the morphology of BMSN was noticed to be almost invariant, however, perfectly spherical shaped particles with regular arrangement were observed.

As per the HR-TEM micrographs, BMSN formed a perfectly spherical shape (Fig. 2h–k) with an average particle size of 262 nm (450–600 °C), where the particle size was almost unchanged. The importance of the BMSN particles dominated by (i) long-range order, and (ii) homogeneity and (iii) smoothness. According to previous results, the particle sizes of chemical templated materials were reported as 200–800 nm,^45^ whereas the size of the obtained BMSN was lower, in the range of 61–300 nm.

Furthermore, HR-TEM-EDXS confirmed the existence of silicon and oxygen in the BMSN. The weight (%) of silicon increased with increasing calcination temperature from 450 °C to 550 °C, then declined at the calcination temperature of 600 °C. This occurred due to the enhancement of the particle diameter at the calcination temperature of 600 °C and this phenomenon was illustrated by FE-SEM and HR-TEM. Hence, the elemental composition (silicon and oxygen) of the synthesized material demonstrated the formation of a MM.

#### 3.1.7 Specific surface area, pore diameter, and pore volume

**Fig. 3:**
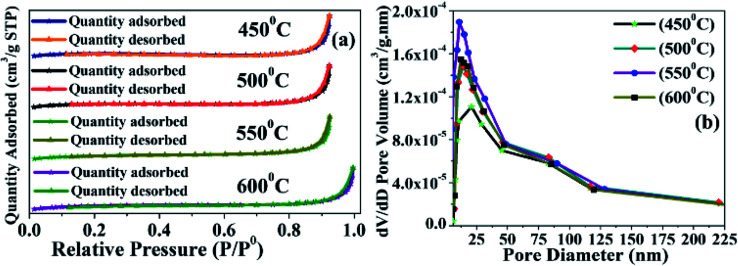
Characterization of synthesized BMSN material calcinated at different temperatures (450 °C, 500 °C, 550 °C, and 600 °C): N_2_ adsorption and desorption (a) and BJH curves (b).

According to IUPAC nomenclature, typical type IV isotherm adsorption was observed in the BET curves^46^ due to the mesoporosity of BMSN (Fig. 3a). The specific surface area and pore volume of the synthesized materials increased with the enhancement of the calcination temperature from 450 to 600 °C (Table 3). The BJH adsorbed average pore diameters of the particles in calcinated at different temperatures are shown in Table 3. The specific surface area of a BMSN particle was detected as 6.5179, 7.7300, 8.2616 and 7.9856 m^3^ g^−1^ consecutively at STP (standard temperature and pressure) for calcination temperatures of 450 °C, 500 °C, 550 °C and 600 °C, respectively (Fig. 3a). In case of adsorption, the adsorbed quantity increased with increasing relative pressure (*P*/*P*^0^) of multilayered adsorption process (Fig. 3a). The first increment of relative pressure (*P*/*P*^0^) was observed in the range of 0.1–0.2 due to capillary condensation of particles. A large amount of adsorption was observed with a sharp hysteresis loop over the relative pressure (*P*/*P*^0^) range of 0.8–1.0 due to uniform distribution of pore size in BMSN particles, where the desorbed quantity was accompanied by the adsorbed quantity. The pore diameter gradually decreased from 29.7070 to 20.2166, 14.8516 and 16.5246 nm with increasing calcination temperature from 450 °C to 600 °C (Fig. 3b). Thus, in the BMSN particles, the surface area and pore volume/diameter were lower (surface area: 8.2616 m^3^ g^−1^; pore volume: 0.020407 cm^3^ g^−1^, and pore diameter: 14.8516 nm) as compared to those in previous reports on chemical template-based MMs (SBA-15 and MCM-41) (surface area: 780–840 m^2^ g^−1^, pore volume: 1.39, 0.86, 265.161 cm^3^ g^−1^, and pore diameter: 7.14, 3.5 and 2.85 nm).^1,47^

**Table 3:** Textural properties of biologically synthesized mesoporous silica nanoparticles (BMSN) calcinated at different temperatures

Sample	*S*_BET_ (m^2^ g^−1^)	Pore volume (cm^3^ g^−1^)	Pore diameter (nm)
MCM-41 (450 °C)	6.5179	0.015763	29.7070
MCM-41 (500 °C)	7.7300	0.017399	20.2166
MCM-41 (550 °C)	8.2616	0.020407	14.8516
MCM-41 (600 °C)	7.9856	0.017394	16.5246

#### 3.1.8 Formation of BMSN mesoporous material

**Fig. 4:**
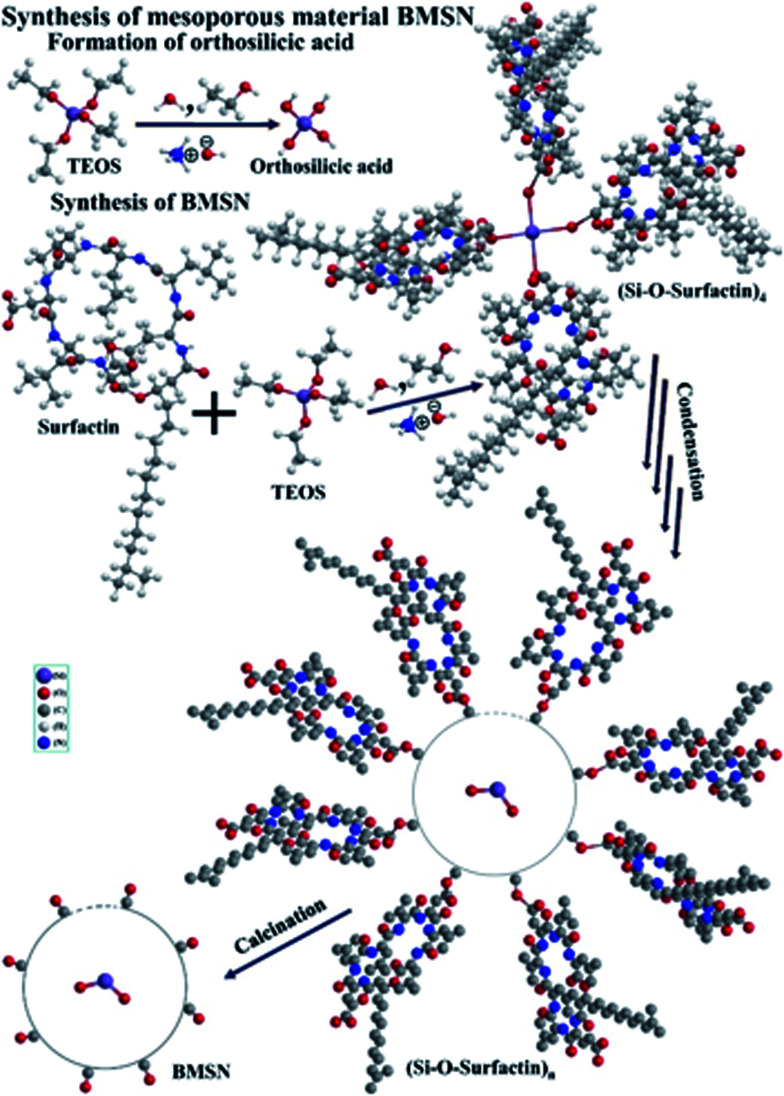
Reaction mechanism for the synthesis of mesoporous material BMSN.

The reaction mechanism in the synthesis of a material is a very important way to understand the tangible formation of particles in material science. The predicted reaction mechanism of BMSN formation is shown in Fig. 4. Initially, the NH_4_OH combines with H_2_O in the presence of C_2_H_5_OH to form NH_4_^+^ and OH^−^ ions by dissociation. The anionic hydroxide further reacts with tetraethyl orthosilicate to produce orthosilicic acid,^48^ where the production of materials takes more than an hour to complete the hydrolysis through a condensation method (Fig. 4). Successively, the biosurfactant combines with tetraethyl orthosilicate in the presence of aqueous NH_4_OH, H_2_O and C_2_H_5_OH, to generate the Si–O–surfactin complexed molecule. Afterwards, in the condensation process, the Si–O–surfactin complexed molecules bind with each other simultaneously and produce a spherical molecule composed of numerous silica–O–surfactin-based complexes as a white powder. Finally, the synthesized material was calcinated to eradicate the biosurfactant from the silica–O–surfactin-based complex [(Si–O–surfactin)_*n*_], forming a highly complexed ordered BMSN mesoporous Si–OH material (Fig. 4).

## 4. Conclusions

A novel BMSN MM was successfully synthesized by an ecofriendly and green process using *Bacillus subtilis* BBK006-mediated biosurfactant as a template using a sol–gel method to diminish the core toxicity in bionanotechnology and clinical studies. The calcination temperature (450–600 °C) influenced the physical and chemical characteristics of the synthesized particles. At the optimum temperature of 550 °C, the crystallographic properties of BMSN confirmed that it was an amorphous silica material with mesoporous character. In the synthesis process, the biosurfactant combines with TEOS (in the presence of NH_4_OH, H_2_O and C_2_H_5_OH) and forms Si–O–surfactin complexed molecules, which later bind with each other and produce a spherical molecule with numerous silica–O–surfactin-based complex through a condensation process. Highly complexed ordered BMSN mesoporous Si–OH material was formed to remove the biosurfactants from the silica–O–surfactin-based complex [(Si–O–surfactin)_*n*_] by calcination. BMSN mesoporous nanoparticles consist of Si–OH, where Si–O–Si linkage is observed. The absorption of BMSN was higher at the wavelength of 256.72 nm in the material calcinated at 550 °C. Effectively, 87.87% yield of BMSN was obtained (weight loss: 12.13%), using the calcination temperature range of 550–600 °C. Perfectly spherical shaped particles with regular arrangement were observed with an average particle size of 280 nm (diameter) (range: 61–300 nm), where the surface area, pore volume and pore diameter were found to be 8.2616 m^3^ g^−1^, 0.020407 cm^3^ g^−1^, and 14.8516 nm, respectively. Thus, this study suggests that materials prepared using biotemplate-based synthesis could be highly efficient and worthwhile for numerous industrial practices with very low toxicity, reduced synthesis time and cost, as well as applications in catalysis, drug delivery systems, gas separation, cosmetics, diagnostics, bio-separation, *etc.*

## Author contributions

R. K. S. prepared the manuscript. J. P. M., J. B., P. G. H., and Y. H. H. revised and gave necessary inputs. J. P. M., C. Y. C., S. C. W., H. C. C., and T. H. C. revised and gave suggestions for the manuscript. J. P. M., G. D., P. B., and S. C. W. revised the manuscript, tables, and figures.

## Conflicts of interest

There are no conflicts of interest.

## Supplementary Material
